# Blood biomarkers of Alzheimer's disease in Australians habitually consuming various plant-based diets

**DOI:** 10.1177/13872877251351549

**Published:** 2025-06-30

**Authors:** Shaun Eslick, Grace Austin, Jessica JA Ferguson, Manohar L Garg, Christopher Oldmeadow, Ralph N Martins

**Affiliations:** 1 Macquarie Medical School, Macquarie University, Macquarie Park, NSW, Australia; 2 School of Biomedical Sciences and Pharmacy, University of Newcastle, Callaghan, NSW, Australia; 3 School of Health Sciences, University of Newcastle, Callaghan, NSW, Australia; 4 Clinical Research Design, Information Technology, and Statistical Support Unit, Hunter Medical Research Institute, New Lambton Heights, NSW, Australia; 5 Edith Cowan University, Perth, WA, Australia

**Keywords:** Alzheimer's disease, biomarkers, diet, plant-based, prevention

## Abstract

**Background:**

Evidence suggests that plant-based diets (PBDs) may be protective against neurodegenerative diseases such as Alzheimer's disease (AD).

**Objective:**

This study examined associations between blood-based AD biomarkers in individuals 30–75 years without current or diagnosed cardiovascular disease following different PBDs versus regular meat-eating diets (RMEs).

**Methods:**

This secondary analysis of the Plant-based Diets study measured Aβ_1–42_/Aβ_1–40_, p-tau181, NFL, and GFAP in 237 plasma samples using SIMOA from individuals following vegan, pesco-vegetarian (PVs), lacto-ovo vegetarian (LOVs), semi-vegetarian (SVs), or RME diets. Multivariable regression adjusted for age and sex.

**Results:**

Following adjustments for age and sex, plasma Aβ_1–42_/Aβ_1–40_ ratio was significantly higher in PVs 0.011 (CI: 0.006, 0.016, p < 0.01), LOVs 0.011 (CI: 0.007, 0.016, p < 0.01) and SVs 0.015 (0.009–0.020, p < 0.01) groups compared to RMEs. Plasma p-tau181 was significantly higher in PVs 3.4 (CI: 0.4–6.4, p < 0.05) and LOVs 7.1 (CI: 2.5, 11.8, p < 0.01), NFL higher in PVs 5.2 (CI: 1.6, 8.7, p < 0.01) and LOVs 4.0 (CI: 1.6, 6.5, p = 0.01), and GFAP higher in PVs 26 (CI: 6, 47, p < 0.05) and LOVs 21 (5, 367, p = 0.01), all compared to RMEs.

**Conclusions:**

This analysis suggests that PBDs may be associated with blood-based AD biomarkers. Higher Aβ_1–42_/Aβ_1–40_ levels in PV, LOV and SV dietary patterns compared to RMEs could indicate lesser amyloid burden, but elevated levels of other AD biomarkers in some PBDs warrant further investigation into nutrient-specific roles in AD pathology.

## Introduction

Over recent decades, interest and consumption of plant-based diets (PBDs) have increased, particularly in western societies, with the perception that PBDs are associated with better health outcomes, a reduced environmental footprint and ethical concerns.^1^ Worldwide market research indicates that the number of people adopting PBDs is significantly increasing.^2,3^

One of the main reasons for consumption of a PBD are potential benefits linked to overall health.^1^ Diets higher in plant foods have been previously reported to be beneficial to lower the risk of obesity, type 2 diabetes, cardiovascular disease, some types of cancer and hypertension.^4^ PBDs are also reported to be protective against neurodegenerative diseases, such as Alzheimer's disease (AD).^4,5^ AD a debilitating neurodegenerative disorder, typified by progressive cognitive decline as a result of neuronal loss and brain shrinkage that ultimately leads to death.^6^ AD pathology is characterized by the accumulation of amyloid-β (Aβ) plaques and hyperphosphorylated tau protein neurofibrillary tangles (NFTs) within the brain, which subsequently lead to brain degeneration and loss of function. AD is the most common form of dementia, representing 50–75% of dementia cases.^7^ Currently there are no curative or effective treatments for AD. Commonly, at clinical diagnosis, considerable and irreversible degeneration of the brain has occurred, therefore early detection and prevention of AD is key to delay the burden and symptoms of the disease.

The development of AD is influenced by non-modifiable risk factors such as aging and genetics, but modifiable lifestyle factors, including nutrition, also play a significant role in AD risk.^8^ A diet rich in healthy fats, antioxidants, vitamins and minerals is crucial to preserve brain and maintain cognitive performance with aging.^9^ Evidence demonstrates that diets that follow principles of the Mediterranean (MeDi) and Dietary Approaches to Stop Hypertension (DASH) diets can decrease cognitive decline and AD risk.^5,10,11^ The Mediterranean-DASH Intervention for Neurodegenerative Delay diet (MIND), has been shown to significantly reduce cognitive decline associated with ageing and decrease incidence of AD.^10,11^ The MIND diet promotes intake of plant-based foods, particularly berries and leafy green vegetables and decreased consumption of animal products and high saturated fat foods. A study by Agarwal et al.^12^ observed that both the MIND and MeDi diets were associated with less amyloid beta load. Previously, cross-sectional evidence has shown that MeDi adherence is associated with reduced AD biomarkers, such as p-tau181 and the AB42/40 ratio in cerebrospinal fluid.^13^ Conversely, a 2018 study by Hill et al.^14^ highlighted that higher consumption of takeaway foods, added sugar, confectionary and cakes, biscuits, and sweet pastries was a significant predictor of amyloid deposition in Australian women. Current literature highlights the importance of nutrition as a modifiable risk factor in the pathogenesis of AD.

Positive effects of PBDs on brain health are reportedly linked to subsequent weight loss, cardiometabolic health improvements and reduced cancer incidence.^15,16^ Compared to regular meat eaters (RMEs), PBDs are typically richer in nutrients reported to have protective effects against AD pathology such as fiber (133–209%), polyunsaturated fats (6.79–8.84%),^16^ as well as vitamins A, B1, B6, C and E as well as folate, magnesium, copper and antioxidant rich compounds such as flavonoids.^17–19^ Meta-analyses show that increased consumption of fruits and vegetables (100 g/day) can reduce dementia risk and delay cognitive decline by 13% in older adults.^20,21^ In contrast, diets that were high glycemic and high in saturated fats were found to be associated with lower Aβ_1–42_ in cerebrospinal fluid (CSF), a marker of AD pathology.^22,23^ Evidence highlights several potential mechanisms for positive AD-related changes linked with PBDs. These include; reduction of systemic inflammation, positive shift in gut microbiota composition and lower chronic disease risk.^24^ Pro-inflammatory cascades can play a significant role in the development of AD. Higher concentrations of inflammatory markers have demonstrated to be associated with cognitive decline in mild cognitively impaired or probable AD individuals.^25^ It is believed that pro-inflammatory cascades contribute to the development of AD when amyloid beta levels are continuously increased through mobilization of the innate immune system via microglia activation.^26^ A systematic review undertaken by Barbaresko et al. suggested that dietary patterns rich in fruits or vegetable were negatively associated with C-reactive protein, a marker of low grade inflammation, highlighting the potential anti-inflammatory benefit of PBDs.^19^ Numerous chronic diseases such as hypertension, type 2 diabetes and obesity have been linked to increased AD risk. Evidence suggests that PBDs have the ability to lower cardiovascular risk, reduce total cholesterol levels, blood glucose and blood pressure, subsequently reducing the risk of these chronic diseases.^15,27–29^ Moreover, among Australians, various plant-based dietary patterns have been reported to be associated with lower body weight, central adiposity, BMI, and other cardiometabolic risk factors compared to RME's.^30–32^ Another mechanism in which PBDs may reduce chronic disease is the alteration of gut microbiome, believed to play a significant role in AD pathogenesis. It has been previously reported that the composition of gut microbiome in AD patients is significantly different to cognitively unimpaired individuals, highlighted by a lesser gut microbiome diversity.^33^ PBDs are believed to promote a larger and balanced diversity of gut microbiota as a result of inclusion of a more varied plant food intake, lower protein and fat intake and higher dietary fiber intake and polyphenols.^18^ Due to the increased dietary fiber intake, significant increases in short chain fatty acids (SCFAs), the most abundant metabolites of the gut microbiota, are proposed to regulate synaptic plasticity, subsequently reducing neuroinflammation and Aβ and Tau AD pathology.^34^

To date, few studies have examined the relationship between diet and blood-based AD biomarkers. Particularly, a paucity of evidence has examined the effect of plant-based diets on AD biomarkers. Previous studies have illustrated lower Aβ_1–42_ and Aβ_1–42_/Aβ_1–40_ ratio as well as increased plasma p-tau181 and GFAP in individuals at all stages of AD (preclinical, prodromal and AD dementia).^35–37^ Similarly, elevated levels of blood-based neurofilament light (NFL) which is associated with neurodegeneration has been observed in both prodromal and AD dementia.^38,39^ Evidence indicates that PBDs are neuroprotective against AD and therefore we hypothesize that AD blood-biomarkers will reflect a lower risk of AD in individuals following plant based dietary patterns compared to those following a RME diet. To address this gap in the literature, the aim of this study is to examine the association between AD biomarkers in individuals following different PBDs compared to regular meat-eating diets.

## Methods

### Study design

This study is a secondary analysis of data collected from the Plant-based Diet Study (PBDS) conducted over the period of November 2021 to March 2023. The research protocol for this study has previously been published.^40^ Briefly, eligible individuals were 30–75 years without current or diagnosed cardiovascular disease (CVD) and consuming one of the following dietary patterns for a minimum of six months. An Accredited Practicing Dietitian screened volunteers over the phone utilizing criteria that assessed habitual weekly intake of meat, seafood, eggs, and dairy to categorize participants into dietary patterns; vegan (nil animal-based foods), lacto-ovo vegetarian (LOV, nil meat, ± eggs, ± dairy), pesco-vegetarian (PV, nil meat, seafood consumption ≥1 per week, ± dairy, ± eggs), semi-vegetarian (SV, meat consumption ≤2 per week) or regular meat-eaters (RMEs) (meat consumption ≥7 per week).^40^ This study was approved by the University of Newcastle's Human Research Ethics Committee (HREC 2020-0195).

Participants completed self-reported questionnaires that examined medical history, age, sex, race, level of education, duration of dietary pattern adherence, occupation and socioeconomic status. Body mass index (kg/m^2^)(BMI) was calculated from height (cm) and weight (kg) collected by a certified clinician.

### Blood collection and measurement of blood-based AD biomarkers

Fasting blood samples were collected in Ethylenediaminetetraacetic acid (EDTA) tubes, centrifuged (Heraeus Biofuge Stratos, Germany, Heidelberg, sourced from Australia), and stored at −80°C prior to analysis. Plasma Aβ_1–40_, Aβ_1–42_, GFAP, and NFL and p-tau181 were measured via Ultra-sensitive Single Molecule array (SIMOA) assay platform (Quanterix). Biomarkers Aβ_1–40_, Aβ_1–42_, GFAP, and NFL were analyzed using the Neurology 4-Plex E kit (QTX-103670, Quanterix, Billerica, MA), calibrators were examined in duplicates and samples in singlicates. The levels of positive controls were assessed for quality control, provided in SIMOA kits. The analytical lowest limit of quantification was, 0.353 pg/mL for Aβ_1–40_, 0.239 pg/mL for Aβ_1–42_, 1.82 pg/mL for p-tau181, 0.635 pg/mL for GFAP, and 0.402 pg/mL for NFL.

### Statistical analyses

Statistical analysis was performed using GraphPad Prism Version 9.0 (GraphPad Software, San Diego, CA, USA) and StataCorp version 18.0 (Stata Statistical Software: Release 17, College Station, TX, USA: StataCorp LP)*.* Normality of data was evaluated via Shapiro–Wilk, where normality was indicated by p-value > 0.05 as well as visually by inspecting histograms, quantile plots, and Q-Q plots. Between group differences participant characteristics were analyzed using One-way analysis of variance (ANOVA) or Kruskal Wallis with Tukey's or Dunn's post hoc tests and categorical data was compared using Fisher's Exact. Statistical significance was determined by p-values < 0.05. Graphical comparison between dietary patterns and blood-based AD biomarkers are presented as figures with data presented as mean ± SD using GraphPad software. Lastly, multivariate regression analysis was conducted to examine comparisons between PBDs and RME adjusted for the effects of confounders, age and sex. Alpha significance levels for models were set at p < 0.05. Due to violations of residual heteroscedasticity, Huber-White standard errors were applied to models.

## Results

### Baseline characteristics of study population

Of the 240 participants recruited from the PBDS, 237 samples were available for analysis from participants recruited into five dietary patterns (Table 1), as previously outlined in the PBD study protocol.^40^ Approximately three in four participants were women (77.5%), the mean age was 54 ± 10 years and majority of participants had a higher education (88.2%). The dietary length of participants on average was 16.5 years and the mean BMI was 24.4 kg/m^2^, the upper limit of the healthy weight range (18.5–24.9 kg/m^2^). Age and dietary pattern length were significantly different between the groups, with the vegan group being the youngest (47.4 years) and followed a dietary pattern for the shortest period (6.9 years). % energy from protein was significantly higher in the RME compared to all the PBDs and % energy from carbohydrates was significantly lower than all PBDs albeit PVs. Sex, higher education, and BMI were all similar amongst groups.

**Table 1.** Characteristics of participants from dietary pattern groups.

	Total sample (n = 237)	RME (n = 47)	VG (n = 47)	PV (n = 47)	LOV (n = 48)	SV (n = 48)	*p*
Females	184 (77.6%)	37 (78.7%)	33 (70.2%)	38 (80.8%)	36 (75.0%)	40 (83.3%)	0.58
Age (y)	54 ± 10	56 ± 10^a^	47 ± 10^b^	56 ± 11^b^	54 ± 10.0^b^	55 ± 8.8^b^	<0.01
Higher education	209 (88%)	43 (92%)	41 (87%)	43 (92%)	41 (85%)	41 (85%)	0.85
Diet							
Dietary pattern length (y)	17 ± 1	31 ± 25^a^	7 ± 8^b^	16 ± 15^b,c^	17 ± 14.0^c^	11 ± 14^b,c^	<0.01
Total KJs	9600 ± 2700	8700 ± 2000^a^	9400 ± 2500^a,b^	11,700 ± 3400^b^	9500 ± 2400^a,b^	9400 ± 2400^a,b^	0.04
% energy from protein	16.4 ± 4.1	20 ± 5^a^	16 ± 4^b,c^	17 ± 3.^b^	15 ± 4^c^	15 ± 3^b,c^	<0.01
% energy from fat	37.3 ± 8.1	38 ± 9	35 ± 9	39 ± 8	38 ± 8	37 ± 7	0.27
% energy from carbohydrates	40 ± 9	35 ± 10^a^	44 ± 8^b^	38 ± 9^a,c^	42 ± 9^b,c^	42 ± 9^b,c^	<0.01
BMI	24.4 ± 4.2	25 ± 5	24 ± 4	24 ± 4	25 ± 4	24 ± 4	0.91
Fat mass (%)	23.1 ± 9.0	25 ± 9	22 ± 9	23 ± 9	24 ± 9	22 ± 10	0.59
Lean mass (%)	44.0 ± 9.2	45 ± 9	47 ± 10	43 ± 8	44 ± 9	43 ± 10	0.22
Physical activity (MET)	5400 ± 5200	5000 ± 3600	5800 ± 4100	4800 ± 4100	7000 ± 8800	4400 ± 3500	0.13
Medication^d^							
HRT (%)	21 (9)	5 (21)	2 (4)	5 (11)	5 (10)	4 (8)	0.72
OHA (%)	2 (1)	1 (1)	0 (0)	0 (0)	0 (0)	1 (2)	1.00
Antihypertensive (%)	15 (6)	4 (9)	1 (2)	6 (13)	3 (6)	1 (2)	0.20
Lipid lowering agent (%)	8 (3)	2 (4)	0 (0)	2 (4)	1 (2)	3 (6)	0.65

BMI: body-mass index; MET: metabolic equivalent of task minutes; HRT: hormone replacement therapy; OHA: oral hypoglycemic agent. Data reported as means ± SD for continuous variables and counts and (percentages) for categorical variables. Continuous data was compared using ANOVA or Kruskal-Wallis, and categorical data was compared using Fisher's Exact. ^a,b,c^ Values within the same row without common superscript letters are significantly different (*p* < 0.05). ^d^Participant currently taking medication/supplement as per medical history questionnaire.

### Comparison of AD blood-based biomarkers in plant-based diets compared to regular meat eaters

#### Aβ_1–42_/aβ_1–40_ ratio

**Figure 1.**
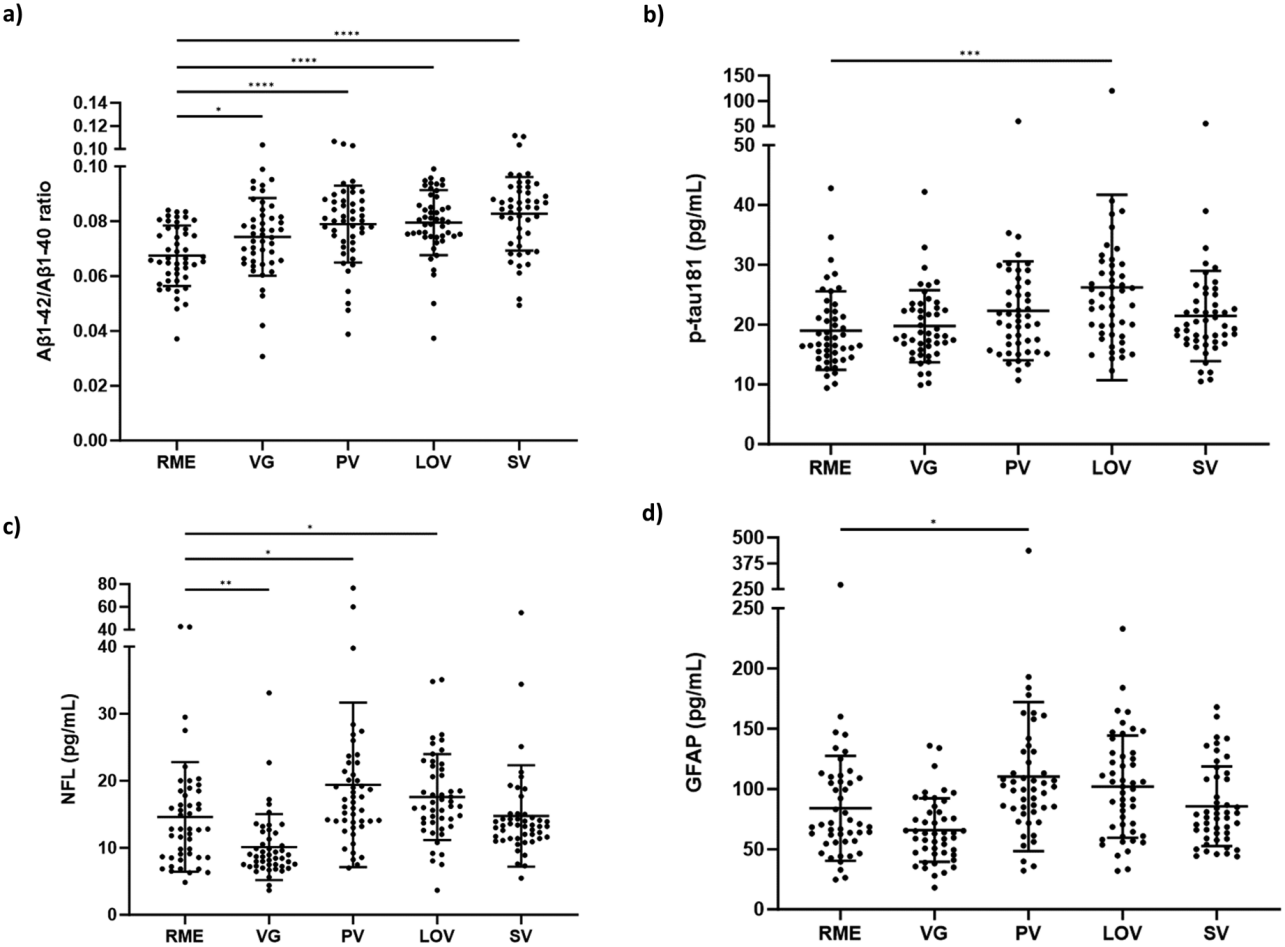
Alzheimer’s disease blood biomarkers sub-grouped by dietary pattern. (a) Aβ_1-42_/Aβ_1-40_; (b) p-tau181 (pg/mL); (c) GFAP (pg/mL); (d) NFL (pg/mL). Aβ: amyloid-β; pTau: phosphorylated Tau; GFAP: Glial fibrillary acidic protein; NFL: neurofilament light; RME: regular meat eaters (n = 47); VG: vegan (n = 47); PV: pesco-vegetarian (n = 47); LOV: lacto-ovo vegetarian (n = 48); SV: semi-vegetarian (n = 48). *p < 0.05, **p < 0.01, ***p < 0.001. Data presented as the mean (SD).

Plasma Aβ_1–42_/Aβ_1–40_ ratio was found to be significantly higher for all PBDs compared to RMEs (Figure 1(a)). Plasma Aβ_1–42_/Aβ_1–40_ ratio was significantly higher in VGs (p = 0.04), PVs (p < 0.01), LOVs (p < 0.01) and SVs (p < 0.01) dietary patterns compared to RMEs.

After adjustments for age and sex, Aβ_1–42_/Aβ_1–40_ ratio (Table 2) was significantly higher in PVs 0.011 (CI: 0.006, 0.016, p < 0.01), LOVs 0.011 (CI: 0.007, 0.016, p < 0.01) and SVs 0.015 (0.009, 0.020, p < 0.01) compared to RMEs. An increasing trend of Aβ_1–42_/Aβ_1–40_ ratio was observed in VGs 0.005 (CI: −0.001, 0.010, p = 0.06) compared to RMEs, however was not statistically significant.

**Table 2.** Multivariable regression analysis of AD blood-based biomarkers across plant-based diets compared to a regular meat-eating diet.

Comparison to RME (n = 47)	Vegan (n = 47)		Pesco-vegetarian (n = 47)		Lacto-ovo vegetarian (n = 48)		Semi-vegetarian (n = 48)	
	β (95% CI)	*p*	β (95% CI)	*p*	β (95% CI)	*p*	β (95% CI)	*p*
Aβ_1–42/1–40_ (pg/mL)	0.005 (−0.001, 0.010)	0.06	0.011 (0.006, 0.016)	**<0**.**01**	0.011 (0.007, 0.016)	**<0**.**01**	0.015 (0.009, 0.020)	**<0**.**01**
p-tau181 (pg/mL)	0.9 (−1.9, 3.6)	0.53	3.4 (0.4, 6.4)	**0.04**	7.1 (2.5, 11.8)	**<0**.**01**	2.6 (−0.2, 5.4)	0.06
NFL (pg/mL)	−0.1 (−2.4, 2.1)	0.90	5.2 (1.6, 8.7)	**<0**.**01**	4.0 (1.6, 6.5)	**<0**.**01**	0.7 (−2.0, 3.5)	0.59
GFAP (pg/mL)	−3 (−16, 11)	0.67	26 (6, 47)	**0.01**	21 (5, 37)	**0**.**01**	2 (−13, 17)	0.77

RME: regular meat eaters; Aβ: amyloid-β; p-tau181: phosphorylated tau; GFAP: glial fibrillary protein; NFL: neurofilament light.

Data presented as β coefficients (95% CIs) and p-values. Multivariate regression analysis adjusted for age (years) and sex (male, female). Values in bold indicate statistical significance (*p* < 0.05).

#### p-tau181

Plasma p-tau181 was significantly higher in the LOV group compared to RMEs (p < 0.01) (Figure 1(b)). p-tau181 was not found to be statistically significantly different between other PBDs and RME.

After adjustments for age and sex, plasma p-tau181 (Table 2) in PV 3.4 (CI: 0.4, 6.4, p < 0.05) and LOV 7.1 (CI: 2.5, 11.8, *p* < 0.01) were significantly higher than in RME.

#### NFL

Plasma NFL was significantly lower in VGs (p < 0.01) compared to RMEs. In contrast, plasma NFL was significantly higher in PVs (p = 0.01) and LOVs (p = 0.01) compared to RMEs (Figure 1(c)).

After adjustment for age and sex, plasma NFL (Table 2) was significantly higher in PVs 5.2 (CI: 1.6, 8.7, p < 0.01) and LOVs 4.0 (CI: 1.6, 6.5, p = 0.01).

#### GFAP

Plasma GFAP was significantly higher in PVs (p = 0.01) compared to RMEs (Figure 1(d)). GFAP was not significantly different between other PBDs and RMEs.

After adjustment for age and sex, plasma GFAP (Table 2) was significantly higher in PV 26 (CI: 6, 47, p < 0.05) and LOV 21 (5, 37, p = 0.01).

## Discussion

The PBD Study was a cross-sectional study performed in Australia to examine cardiovascular health and dietary profile of PBDs among Australians purposefully sampled for their habitual PBD adoption. This secondary analysis represents a novel investigation of blood-based AD biomarkers in individuals habitually following different PBDs compared to RMEs. This analysis demonstrated that significant differences in AD biomarkers Aβ_1–42_/Aβ_1–40_ ratio, p-tau181, GFAP and NFL exists amongst individuals following various PBDs. Dietary patterns, independent of age and sex, may be associated with expression of blood-based AD biomarkers. Higher plasma Aβ_1–42_/Aβ_1–40_ ratio that is known to be associated with reduced risk of AD, after adjustments was significantly higher in PVs, LOVs and SVs compared to RMEs. In contrast, after adjusting for age and sex, higher p-tau181, NFL and GFAP were observed between PVs and LOVs compared to RMEs, where elevation of these biomarkers is linked with increased risk of AD. The results from this study indicate that some PBDs may be associated with expression of AD biomarkers.

The deposition of Aβ in the brain is a typical neuropathological alteration that can occur up to 30 years prior to the clinical and diagnosis of AD.^41^ High levels of Aβ in cerebrospinal fluid or plasma and low deposition within the brain signify a normal Aβ status.^42^ Previous evidence highlighted the association between lower Aβ_1–42_/Aβ_1–40_ ratio and higher amyloid cortical burden and accelerated amyloid accumulation, greater cognitive decline, and increased risk of Alzheimer's disease development.^43–45^ In contrast, evidence has indicated that western diets, typically high in saturated fat and refined carbohydrates can exacerbate Aβ peptide concentration and subsequently result in a higher risk of dementia.^46^ In this study, analysis revealed that Aβ_1–42_/Aβ_1–40_ ratio was significantly higher in individuals following PV, LOV, and SV plant-based diets after adjusting for age and sex compared to RME. Additionally, Aβ_1–42_/Aβ_1–40_ trended higher in the VG group compared to RME (p = 0.06). A paucity of data has investigated the effects of diet on AD biomarkers, particularly blood-based biomarkers. Similar to our findings, previous evidence has demonstrated, that both mediterranean and MIND diets have been associated with lesser global AD pathology (neuritic plaques, diffuse plaques and neurofibrillary tangles) and less Aβ load, even after controlling for other cardiovascular factors such as physical activity, smoking and vascular disease burden and in various population studies.^13,47^ Similar to plant-based diets, the mediterranean and MIND diets, both highlight the importance of fruits, vegetables and wholegrains and lower consumption of red meat, refined carbohydrates and processed nutrient-poor foods. Our findings in conjunction with current literature may suggest that plant-based diets are associated with a lower risk of amyloid accumulation and subsequently a reduced risk of Alzheimer's neuropathology. Future studies that examine AD biomarker expression in individuals following PBDs with cognitive impairment or pre-clinical Alzheimer's disease may further elucidate relationships between diet and AD pathology.

In contrast to AB42/40 ratio findings, observations amongst other AD blood-based biomarkers in this study were not as clear. Increased plasma p-tau181 has been previously associated with increased global Aβ and tau burden as well as cognitive decline.^48^ After adjusting for age and sex, p-tau181 was significantly higher in both PV and LOV groups compared to RMEs. Whilst no studies have investigated the impact of PBDs on plasma p-tau181, previous evidence has shown that a western dietary pattern was associated with preclinical AD, specifically Aβ_1–42_ and t-Tau and/or p-tau181 pathology.^49^ In the same study, a western dietary pattern was significantly associated with T-tau alone however not Aβ_1–42_/Aβ_1–40_ or p-tau181 biomarkers alone.^49^ Our Aβ_1–42_/Aβ_1–40_ findings suggested a lower AD risk in those following PBDs compared to RME, however the same results were not seen in p-tau181. Previously, a study by Ballarini et al. examined the effect of the MeDi diet on CSF AD biomarkers and found that higher adherence to MeDi was significantly associated with lower levels of CSF p-tau181.^13^ Findings between p-tau181 in our study have not aligned with those seen in previous literature, perhaps due to age, where the mean age of the PBD study (53.6 years) is significantly lower than that of other studies (>60 years), in which p-tau181 and diet has been investigated. Additionally, inconsistency between p-tau and Aβ findings within this study may be further explained by the hypothesized order of neuropathological events in AD. Evidence suggests that AD neuropathology begins with deposition of Aβ, then led by the formation of neurofibrillary tangles caused by hyperphosphorylated tau.^50^ A study by Luo et al.^51^ examined the sequence of AD biomarker changes in cognitively normal individuals and found that changes in CSF AB42/40 can appear as early as approximately 46 years, whilst CSF p-tau181 had a change point of approximately 60 years of age. These findings supported the hypothesis that amyloid changes can occur at an earlier age compared to p-tau changes. The mean age of participants within this study was 54 years old. It is worth noting that p-tau was within healthy reference ranges, perhaps also limiting findings.^52^ Inconsistencies may suggest it is reasonable that markers of amyloid neuropathology are more relevant than p-tau181 in relatively younger populations.

Circulating levels of plasma biomarkers GFAP and NFL have previously been shown to reflect AD-related neuropathology.^53^ A study by You et al.^54^ conducted proteomic profiling on approximately 52,000 plasma samples from the UK biobank and found that proteins GFAP and NFL were consistently associated with all all-cause dementia, Alzheimer's disease and vascular dementia. Fewer studies have examined the effect of diet on these biomarkers. Similar to p-tau181 findings, NFL and GFAP were significantly higher in PV and LOV groups compared to RME, following adjustments for age and sex. One study has examined the effect of specific foods on plasma AD biomarkers in individuals along the AD continuum. Findings showed that red meat, grains, and beans were associated with higher levels of plasma NFL.^55^ These might suggest that specific foods in different diets may impact NFL levels. Similar to p-tau181, NFL and GFAP were also considered to be within healthy reference ranges in the current study, perhaps diminishing the findings. With few studies examining the effects of specific diets and foods on NFL and GFAP blood biomarkers, future research should investigate these further, to elucidate possible roles between nutrient intake and NFL and GFAP expression and establish an understanding of the interplay between dietary intake and their expression.^43–45^

There are a few limitations in this study to acknowledge. Firstly, the mean age of this sample is slightly younger (54 years) than other studies examining diet-disease AD pathology, where mean age is often around 60 years.^22,23^ However, significant research has indicated that presence of AD pathology can precede clinical AD by up to 20–30 years, providing justification for exploring AD biomarkers in this study.^56^ Further, evidence may highlight the importance of following plant rich diets in the earliest stages of AD pathology. Secondly, this sample may have been at risk of selection bias, as individuals who volunteered may have higher education, higher socioeconomic status, or health motivation. As with most observational studies, this is a factor that cannot be controlled. Lastly, as this study is a cross-sectional study, causal inference cannot be made between diet and AD biomarkers. Despite some limitations, this novel study provides insight into pre-clinical AD pathology in Australian individuals following various PBDs compared to RMEs. A further strength of this study is the purposeful recruitment of individuals following various PBDs for at least 6 months and dietary screening by a trained dietitian.

### Conclusion

To date, few studies have examined diet and AD blood-based biomarkers. This novel study examines blood-based AD biomarkers in several PBD dietary patterns in comparison to a typical regular-meat diet. Findings from this study indicate that PBDs may be neuroprotective against AD pathology compared to regular meat-eating diets, as evidenced by the reduction in Aβ_1–42_/Aβ_1–40_ ratio between PBDs and RMEs. However, results were conflicting, as other AD biomarkers were significantly higher in some PBDs compared to RMEs. Future studies should attempt to examine findings in larger studies to strengthen the observable power of analysis, as well as explore potential associations between specific nutrients within such dietary patterns and AD biomarkers. Future studies may also benefit from investigating diet and AD biomarkers in older adults or those in different stages of AD to understand whether greater differences exist between expression of biomarkers due to presence of greater disease pathology.
